# Cushioning mechanism of the metatarsals during landing for the skateboarding ollie maneuver

**DOI:** 10.3389/fbioe.2024.1382161

**Published:** 2024-04-22

**Authors:** Yusen Wu, Haichun Wang, Cheng Deng, Yangyu Guo, Xiaolan Zhu

**Affiliations:** Sport Science School, Beijing Sport University, Beijing, China

**Keywords:** multibody coupling, skateboard, landing, biomechanical mechanism, finite element method

## Abstract

Skateboarding is an Olympic event with frequent jumping and landing, where the cushioning effect by the foot structure (from the arch, metatarsals, etc.) and damping performance by sports equipment (shoes, insoles, etc.) can greatly affect an athlete’s sports performance and lower the risk of limb injury. Skateboarding is characterized by the formation of a “man–shoe–skateboard system,” which makes its foot cushioning mechanism different from those of other sports maneuvers, such as basketball vertical jump and gymnastics broad jump. Therefore, it is necessary to clarify the cushioning mechanism of the foot structure upon landing on a skateboard. To achieve this, a multibody finite element model of the right foot, shoe, and skateboard was created using Mimics, Geomagic, and ANSYS. Kinetic data from the ollie maneuver were used to determine the plantar pressure and Achilles tendon force at three characteristics (T1, T2, and T3). The stress and strain on the foot and metatarsals (MT1–5) were then simulated. The simulation results had an error of 6.98% compared to actual measurements. During landing, the force exerted on the internal soft tissues tends to increase. The stress and strain variations were highest on MT2, MT3, and MT4. Moreover, the torsion angle of MT1 was greater than those of the other metatarsals. Additionally, the displacements of MT2, MT3, and MT4 were higher than those of the other parts. This research shows that skateboarders need to absorb the ground reaction force through the movements of the MTs for ollie landing. The soft tissues, bones, and ligaments in the front foot may have high risks of injury. The developed model serves as a valuable tool for analyzing the foot mechanisms in skateboarding; furthermore, it is crucial to enhance cushioning for the front foot during the design of skateboard shoes to reduce potential injuries.

## 1 Introduction

Skateboarding is a skill-based sport that requires the players to perform stunts in a designated venue, including slides, jumps, and rotations (Dickinson et al., 2021). The difficulty of the maneuvers mainly affect the sports item scores, and the Paris 2024 Olympic Skateboarding Rules note that two-thirds of the points are to be decided based on the performed tricks (Depasse, 2022). Ollie is a beginner maneuver with less skill and difficulty among all maneuvers; however, it still exerts an impact force of approximately 3.77 body weight (BW) on the human body and especially the lower limbs, even though skateboarders land on the ground upon successful completion of an ollie, with the skateboard attenuating a part of the reaction force (Nevitt et al., 2008). Injuries of the lower limbs have been reported frequently (69.7%) in research on skateboarding damage, with ankle injuries notably being the most common (26.4%), which may be related to carrying such an immense impact (Feiler and Frank, 2000; Rodriguez-Rivadulla et al., 2020; Ferreira et al., 2021).

Ollie has a focal point in skateboarding because of its representativeness of low-level stunts among all skateboarding maneuvers (Vorlicek et al., 2015; Leuchanka et al., 2017). Previous studies have reported the kinematic, kinetic, and EMG data of ollie using inertial measurement units (IMUs), force plates, in-shoes pressure sensors, and surface electromyography (Frederick et al., 2006; Groh et al., 2015; Vorlicek et al., 2015; Leuchanka et al., 2017). In addition to technique advancing, the multibody dynamics model was used to detect ollie’s jumping actions with the purpose of studying the relationship between the board tail, rear wheels, and foot (Nakashima and Chida, 2021). However, current research on skateboarding is insufficient and lacks a good understanding of how the human body is buffered in the process of skateboarding landing. Understanding this mechanism can help skateboarders in preventing injuries and manufacturers with designing appropriate equipment.

Absorption of the collisional energy and action of a buffering structure are crucial for both uncomplicated walking movements and other sports with repeated landing maneuvers, such as running (Yang, 2018), volleyball (Farzami and Anbarian, 2020), basketball (Belcher et al., 2022), and gymnastics (O'Kane et al., 2011), as previous studies have reported that shock attenuation can be realized by changing the shapes of the longitudinal and transverse arches (Zhang et al., 2018; Bai and Huo, 2022). An in-depth analysis of the shape alteration of the foot arch structure has also been conducted, which is mainly attributed to rotation and displacement of the metatarsals (MTs), contributing greatly to energy absorption and force transformation (Olsen et al., 2018; Nakashima and Chida, 2021). The foot arch performance and adjustments of the rotation angle and displacement of the MTs have certain similarities in the aforementioned sports; however, these are still underreported for skateboard landing, so detailed research under rigorous considerations is of great necessity. The skateboarding field includes bowls, railings and stairs (Dickinson et al., 2021), and its environment is more complex than that of gymnastics (O'Kane et al., 2011), basketball (Belcher et al., 2022), or other sports. Furthermore, there are many conductive media involved in the landing process of a skateboard, such as feet, shoes, board, wheels, and the ground, so that the force conduction is more complex. Consequently, it is necessary to analyze the landing mechanism in skateboarding.

Markers and inverse dynamics are frequently used to obtain kinematic data on the small bones of the foot, such as metatarsals, cuneiform, and talus, in the laboratory testing environment (Kirby, 2000; McPoil et al., 2009). Nevertheless, an indirect inference of bone movements may present significant uncertainties because the markers are often affixed at the bone counterparts on shoes. Contrary to traditional kinematic and kinetic data acquisition, finite element (FE) analysis offers the advantage of detecting the motions and stress–strain conditions of the object’s internal structure; it has therefore been widely adopted to explore the ground contact mechanics of running shoes, soft tissues, and internal stress–strain simulations of bones, etc. (Zhang et al., 2007; Song et al., 2022b). In addition, the FE method can be used to speed up the design cycle while reducing research and development costs in the design of sports equipment, such as carbon-fiber plates (Song et al., 2023), running shoes (Song et al., 2022a), midsoles (Zhu et al., 2023), and insoles (Shi et al., 2022).

Accordingly, FE analysis was used in this study to investigate the internal movements and landing collision mechanism of the MTs in the ollie landing motion. Prior to formal simulation of the ollie landing, a mutibody coupling FE model of the skateboard, right foot, shoes, and ground was established. Based on previous investigations and reports, the following were hypothesized: First, the error rate between the FE simulation result and experimental testing outcome for stationary standing on a skateboard was less than 10%. Second, the MTs were found to play critical roles in energy absorption and shock attenuation during the ollie landing process, especially the middle parts of the MTs, which are the main weight bearers.

## 2 Materials and methods

**FIGURE 1:**
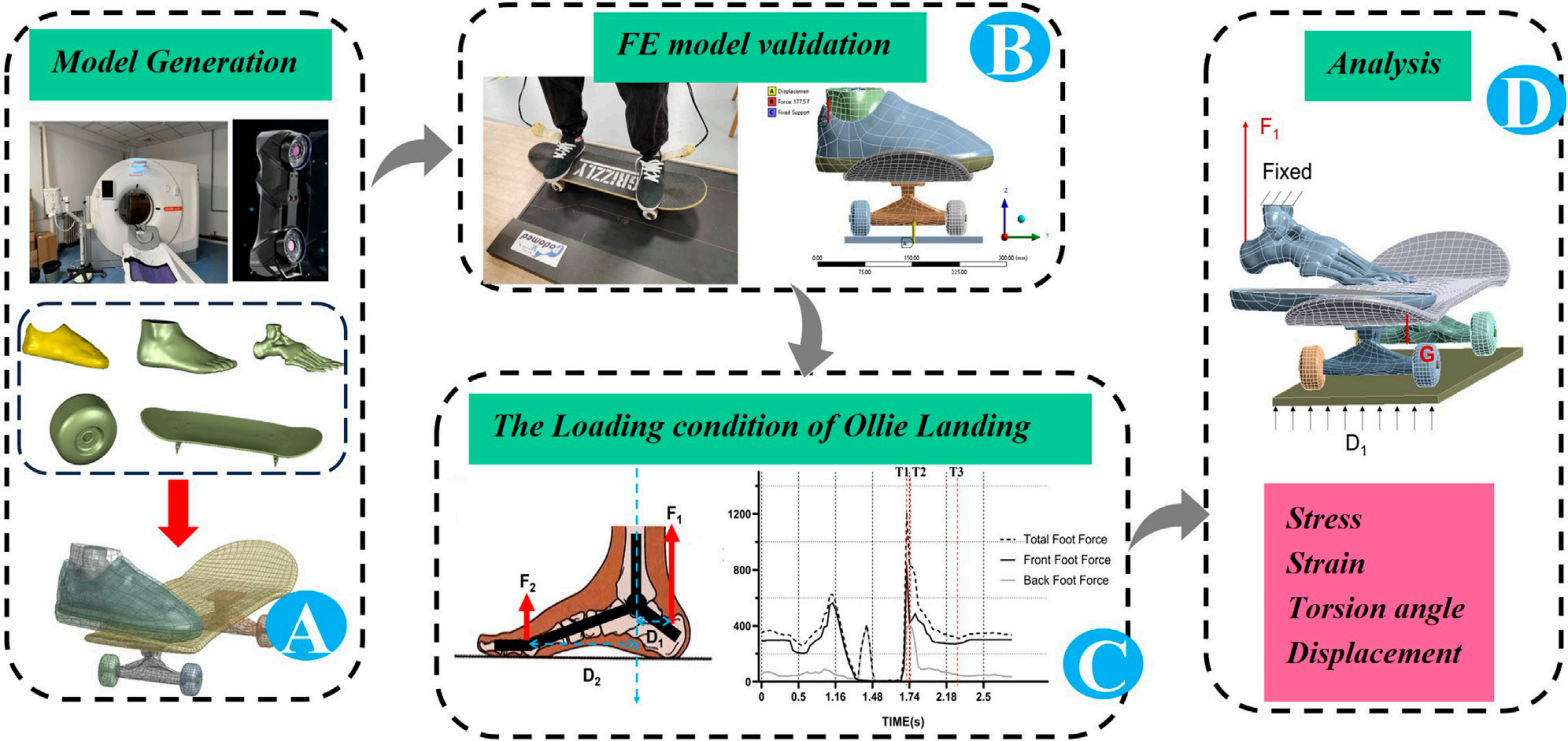
Finite element (FE) model simulation process: **(A)** The establishment of finite element (FE) model; **(B)** Verification of the FE model; **(C)** Simulation of Ollie landing; **(D)** The output of the calculation results.

A 24-year-old male skateboarder, with a height of 178 cm and weight of 71 kg, was recruited for this study. The foot length (i.e., the maximum straight-line distance from the heel of the foot to the longest toe) and foot width (i.e., the linear distance from the first to fifth MTs) were measured to be 26 cm and 10.4 cm, respectively. The ES skateboard shoes (ES, Sole Technology Company, United States) were adopted as the shoes for the experiment. The program was approved by the Ethics Committee of Beijing Sport University (2023143H), and the participant read and signed the informed consent before data collection. The upper material of the shoe is suede, and the outsole is made of rubber. An 8.0-inch street skateboard was used as the experimental skateboard, which includes the Deathwish deck, Theeve’ Titanium TIAX bridge, and Bones STF 99A V1 wheels (Figure 1A). Scanned models of the subject’s foot, skateboard shoe, and skateboard were acquired using a CT scanner (Siemens SOMATOM go, Top 64-row 128-slice spiral CT machine, Berlin, Germany) and a 3D laser scanner (Handyscan Black Elite, Creaform, Levis, Quebec, Canada). Model extraction and restoration were performed using Mimics 21.0 (Materialise Inc., Leuven, Belgium) and Geomagic Studio 2013 (Triangle Development, North Carolina, United States). A flat insole (front thickness: 4 mm, back thickness: 6 mm, waist height: 10 mm) model with a reference to the scanned foot and a level ground (75 cm × 35 cm × 1 cm) for the simulation were obtained using SOLIDWORKS 2022. The model coupling was done in ANSYS 2020R2 (Swanson Analysis, Houston, Pennsylvania, United States) (Figure 1A). The FE model included one set of bones of the right foot (tarsals, MTs, phalanges, distal tibia, and distal fibula), one set of soft tissues, one upper shoe, one outsole, one insole, one deck, two bridges, four wheels, and the ground.

The FE model was set to an isotropic and uniform linear elastic material (Tongbo et al., 2021). The bridge was made of Ti-6Al-4V provided by the ANSYS material library. More details about the materials of the model are given in Supplementary Table S1. “Face to face” contact was employed for each entity, and two contact types were allocated between the different bodies: the first one between the soft tissues, insole, outsole, and deck was frictional contact with a coefficient of friction of 0.6; the second one between the bones and soft tissues, sole and upper part of the shoe, and parts of the skateboard was bonded contact. For the meshing, the tetrahedral method was used to combine automated meshing generation and manual control of the meshing size. The overall unit sizes of the bones and soft issues are 4 mm each, and the overall unit sizes for the shoe and board are 7.0 mm each. To improve the accuracy of analysis, local mesh refinement of the contact areas of the foot, shoe, and skateboard was performed. In addition, sensitivity analysis was conducted based on the peak plantar pressure to balance the computational efficiency and solution accuracy of the model. The meshing quality was 0.79, with a total of 408,043 nodes and 260,400 elements.

### 2.1 Landing and boundary conditions

A static standing simulation was carried out using the FE model. In the standing state, each foot bears one-half of the gravitational force (Bocanegra et al., 2021), and only the triceps of the calf has an obvious EMG signal (Liu et al., 2022). Studies show that the force of the Achilles tendon is 50% of the force of the entire foot (Sun et al., 2020). Therefore, the Achilles tendon force in this study was set at ¼ (177.5 N) of the subject’s gravity. In the actual test, the body–skateboard system would have a downward displacement, but this value was difficult to obtain. There are some limitations to measuring the vertical downward displacement data of the human–skateboard–ground system during standing. Therefore, this study simulates the downward displacement of the overall system in the standing state by fixing the upper surfaces of the tibia and fibula, adding the vertical upward displacement by the floor, and restricting the front-to-back and left-to-right displacements. The optimal displacement of 1.4 mm was obtained by repeatedly adjusting the amount of ground upward displacement (Figure 1B). The model error was defined as the percentage difference between the simulated value and measured result with respect to the measured result.

### 2.2 Experimental validation

The model was verified by comparing the plantar pressure of the simulation with the measured value of the natural standing condition. The data were collected using the Pedar system (PEDAR-X; Novel, Inc., Germany) and Sensor Medica (SENSORMEDICAS.R.L., Italy) as the plantar pressure and pressure of the skateboard wheels. The subject stood naturally on their feet at the bridge peg on the deck, and the test time was 10 s to obtain the peak plantar pressure and peak skateboard pressure. The sole of the foot and bottom of the skateboard are divided into several areas. The sole of the foot is divided into the medial front foot, lateral front foot, middle foot, and heel foot. The bottom of the skateboard is divided into front and rear wheels. Previous studies have reported that the model can be considered effective if the error is less than 10% (Akrami et al., 2018). The interclass correlation coefficient (ICC) was used to assess the consistency between the simulated and test values. The correlation coefficient was defined as |r| ≤ 0.5, indicating poor consistency; 0.5 ≤ |r| ≤ 0.75, indicating medium consistency; 0.75 ≤ |r| ≤ 0.9, indicating large consistency; and |r| > 0.9, indicating good consistency (Koo and Li, 2016). The Bland–Altman plot was used to determine the bias and consistency limits between the methods. SPSS26.0 software (SPSS, Chicago, IL, United States) was used for the consistency analysis, and the two approaches were considered to have good consistency if the difference between them was within the 95% limit of agreement (LOA).

### 2.3 Simulation analysis of ollie landing

The plantar impact data of the foot CT of the model were collected via the Pedar system at a data collection frequency of 50 Hz. To obtain feasible data, the subject performed the ollie movement ten times, with an average jump height of 33.7 cm ± 4.2 cm for each jump. The force–time (F-T) curve of the ollie movement was saved in the MVA format. After smoothing the F-T curve by removing noise directly, three characteristics were found (Figure 1C): the moment of touching the ground (T1), the moment of the peak of the plantar force (T2), and the moment of the trough of the plantar force (T3). T1 corresponded to the initial contact between the skateboard and ground. When the total weight of the skateboarder was entirely on the skateboard, the force increased rapidly to the impact peak, which was T2. T3 occurred when the skater completed buffering and returned to a stable state (Frederick et al., 2006; Determan et al., 2010). In the test results, the peak value of the sole force of the subject’s forefoot was 830.26 N, and the total force of both lower limbs was 1210.63 N (1.74 body weight (BW)), which was similar to the research results of Leuchanka et al. (2017).

The Achilles tendon tension was calculated according to the principle of moment equilibrium (Chatzistergos et al., 2017) (Figure 1C). The Achilles tendon tension was defined as the ratio of the plantar flexion moment of the ankle joint to the Achilles tendon lever. The plantar flexion moment was the product of the maximum plantar force and flexion lever. The flexion lever was simplified as the sagittal distance between the position of the maximum plantar force and center of the ankle joint; the distance was output from the plantar pressure data and derived from the coordinates of the pressure peak occurrence. The Achilles tendon lever was simplified as the horizontal distance between the point of Achilles tendon attachment and center of the ankle joint, which was obtained from the CT image using the MIMICS 21.0 measuring tool. The ankle flexion moment and Achilles tendon force are shown in Supplementary Table S2 (the vertical axis joint force is positive in the upward and negative in the downward directions).

$$\sum M=F_{power}L_{power\;arm}+F_{resistance}L_{resistance\;arm}=0.$$

The Achilles tendon forces (Supplementary Table S2) were applied through joint forces. The upper parts of the soft tissues, tibia, and fibula were fixed. A vertical downward displacement was added to the toe tips to simulate toe flexion. The vertical upward displacement was exerted in the geodetic coordinate system so that the plantar pressure reached the peak value, and the displacement of the floor in the geodetic coordinate system was constrained. A 2.223 kg center of mass was added to the skateboard to simulate the effect of gravity on the skateboard (Figure 1D).

## 3 Results

### 3.1 FE modeling and validation results

**FIGURE 2:**
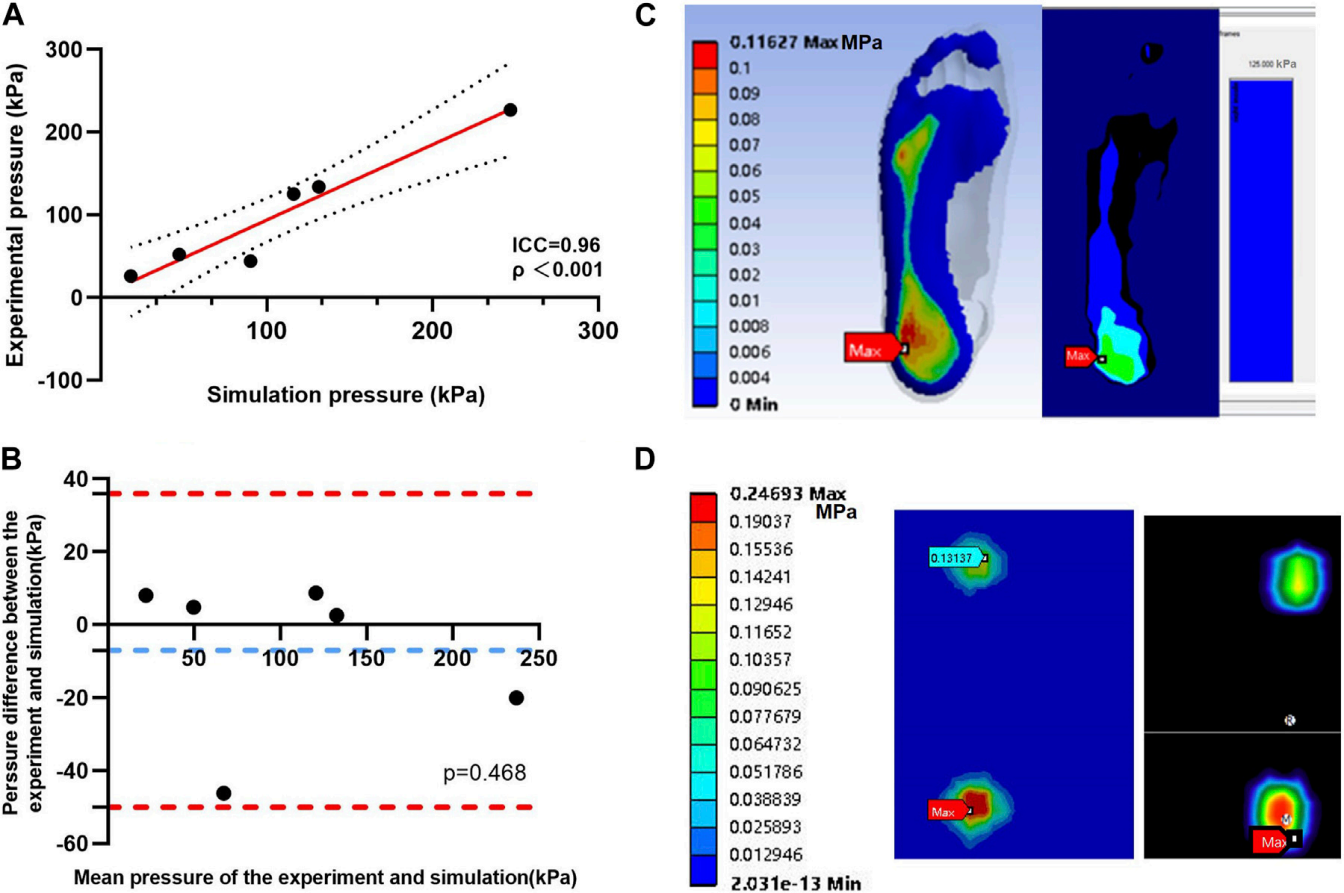
**(A)** Validation of the foot–shoe FE model with experimental pressure using interclass correlation coefficient analysis, and the results are illustrated by the regression line (solid red line) between the predicted and experimental pressures. The black dots represent the 95% confident interval. **(B)** Bland–Altman plot results are illustrated by the differences between pairs of pressures as functions of the mean pressure. The solid blue line depicts the bias, and the red dots are the 95% limits of agreement. **(C)** Plantar pressure data. **(D)** Skateboard bottom pressure data.

The predicted results showed that the peak value of the plantar pressure in the standing state was 116.27 kPa (peak value was in the heel), and the peak value of the skateboard wheel bottom pressure was 246.93 kPa (peak value was in the rear wheel). The predicted results are consistent with the measured results, with an error of about 6.98% for the measured peak pressure (sole: 125 kPa, wheel bottom: 226.93 kPa) at the peak position (sole: heel, wheel bottom: rear wheel). As shown in Figure 2, the ICC test shows an excellent score (0.96) with a 95% confidence interval of 0.86–0.99. The Bland–Altman plot shows that the mean offset was 7.028 kPa, which was not statistically significant (*p* = 0.468). The verification results show that the predicted results of the model are reasonable.

### 3.2 Soft tissue stresses at the three moments during ollie landing

**FIGURE 3:**
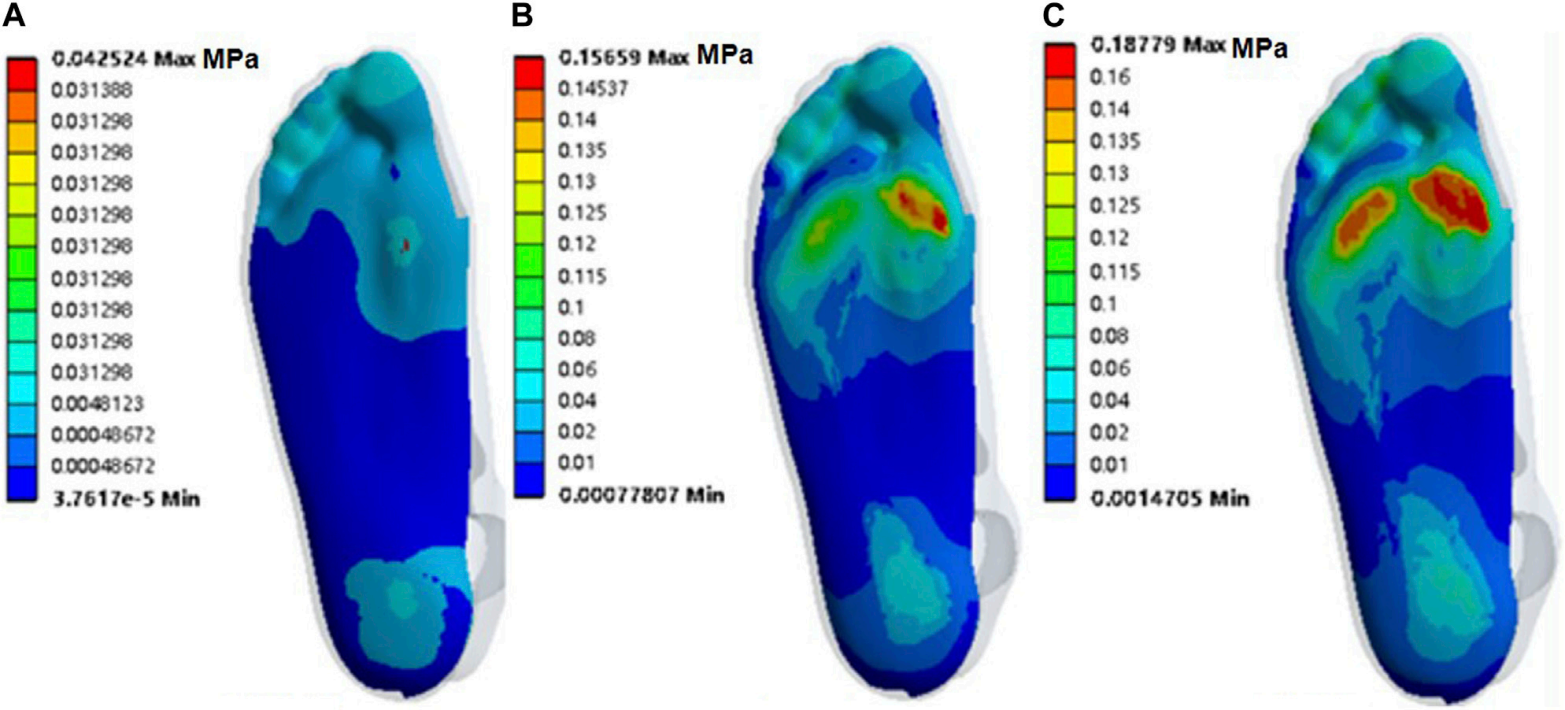
Stresses on the soft tissue during landing: **(A)** T1, **(B)** T2, and **(C)** T3 moments.

During ollie landing, the soft tissue stress is concentrated on the inside of the forefoot, and it gradually increased to reach the maximum stress at T3; the maximum stresses at T1, T2, and T3 were 42.524 kPa, 156.59 kPa, and 187.79 kPa, respectively (Figure 3).

### 3.3 Stresses and strains on the MTs

The largest values of both stress peak (1.126 MPa) and elastic strain (1.548%) at T1 occurred in MT3. The stresses of MT4 were highest at both T2 and T3, peaking at 23.211 and 29.161 MPa, respectively. The elastic strains of MT4 at T2 and T3 were also maximum at 31.803% and 39.956%, respectively (Supplementary Table S3). The changing trends in the peak stresses and strains of the MTs were similar, namely, the tendencies of MT1 to MT5 gradually increased from T1 to T3, eventually peaking at T3.

**FIGURE 4:**
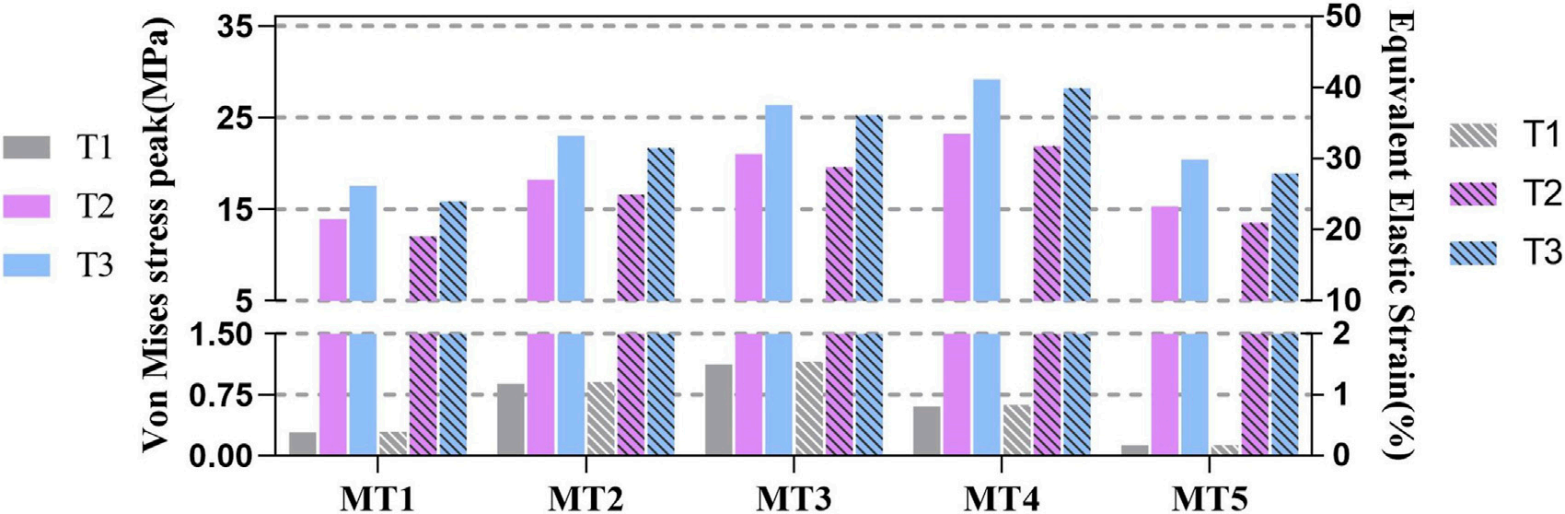
Histograms of the peak stresses and strains in the first to fifth metatarsals at three moments: (T1) the moment of the foot touching the ground; (T2) the moment of the peak plantar force; (T3) the stability moment of the first to fifth metatarsals (MT1-5).

The stress of MT3 was the highest (1.126 MPa) at T1, which was nearly nine times that of MT5 (0.127 MPa). The stresses and strains of the MTs with MT3 as the center decreased gradually on both sides. As the landing proceeded, the body of the maximum stress switched from MT3 (21.032 MPa) to MT4 (23.211 MPa), and the stress of MT5 (15.317 MPa) was no longer the lowest as it was diverted to MT1 (13.928 MPa). MT4 constantly withstood the largest stress (29.161 MPa) among all the MTs, with the stress on MT1 being the smallest (17.549 MPa). Comparing the three characteristics, the amounts of stress on the MTs surged rapidly from T1 to T2 and then slowly added up to T3 (Figure 4).

### 3.4 Torsion angles and displacements of the MTs in the sagittal plane

**FIGURE 5:**
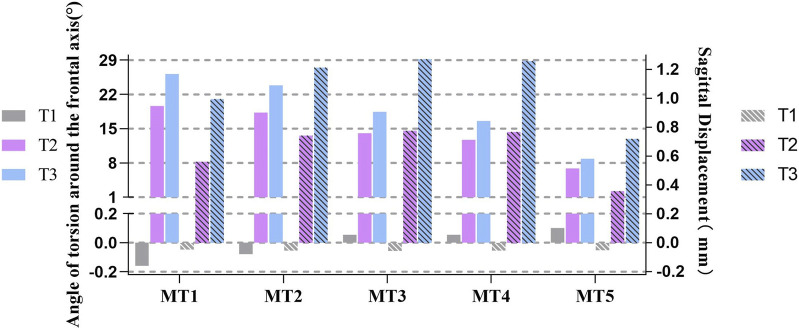
Histograms of the torsion angles and displacements of the metatarsals along the sagittal plane at three moments: (T1) the moment of the foot touching the ground; (T2) the moment of the peak plantar force; (T3) the stability moment of the first to fifth metatarsals (MT1-5) (– toe flexion, + dorsiflexion).

During landing, the torsion angles of the MTs in the sagittal plane varied dramatically (Figure 5), and the continuous increase in terms of this parameter was similar to those from T1 to T3. Moreover, among all the MTs, the variation for MT1 was the sharpest, touching the minimum value of −0.160° at T1 and maximum of 26.181° at T3. Nevertheless, the amplitude change for MT5 was the least, which varied from 0.101° at T1 to 8.864° at T3.

The torsion angles at the three moments represent different meanings (Figure 5). First, toe flexion occurred at MT1 and MT2 on the inside of the frontal foot due to contact between the skateboard and inner foot at T1; the outside of the forefoot did not contact the skateboard, so that dorsiflexion occurred in MT3, MT4, and MT5 due to inertia. Second, the MTs were all twisted in the direction of dorsiflexion, especially the medial MTs. Finally, MT4 and MT5 also experienced large twists (18.452° and 16.594°) at T3. The twisting angles of MT1 and MT2 changed greatly.

Sagittal displacements of the MTs at the three moments mean that the process of landing has different cushioning procedures (Figure 5). At T1, the displacements of all MTs are small; MT1 and MT2 show toe flexion, while the remaining MTs show dorsiflexion. However, the displacement at T2 turned positive, peaking at 0.777 mm on MT3, and the value at T3 increased constantly based on T2, which was 1.272 mm at MT3. These fluctuating trends of the MTs present peaks for T2 and T3. When cushioning, the sagittal planes of MT2, MT3, and MT4 are shifted more; the displacement of the medial MT1 was greater than that of the lateral MT5.

## 4 Discussion

In this study, the impact of collision of a multiple-model system including detailed foot structures, skateboard models, material properties, and experimentally obtained loading conditions was investigated. The error of peak pressure between actual measurement and model simulation is 6.98%. Previous studies have reported that a model can be considered effective if its error is less than 10% (Akrami et al., 2018). However, the distribution plot shows that there are differences in the forefoot; the stress of the forefoot in the simulation is greater than the measured result. The reason for this may be that the angle between the foot and ground is not obtained accurately. The flat foot in the model increases the pressure on the front foot. Herein, CT and 3D scans were used to obtain the images, so the model was more consistent with the human body and its geometric features, with similar mechanical distribution and a low error rate. Therefore, the model established in this study is effective and supports further research.

In this study, it was found that the midfoot MTs primarily played weight-bearing roles in the cushioning process, with the MT1 and MT5 playing secondary roles. Previous studies on jumping in basketball (Belcher et al., 2022) and volleyball (Farzami and Anbarian, 2020) showed that repeated landing movements increase the maximum pressure on the sole of the foot. At the same time, fatigue can lead to lower limb pain and foot damage. The characteristics of the front foot in the ollie landing are consistent with these observations. The increased force on the forefoot and repeated displacement of the arch may lead to risk of damage to the soft tissues, fascia, and ligaments. Being aware of the biomechanics of the foot during ollie landing is thus important for injury prevention and manufacturing supportive equipment. There are a variety of cushion structures in skateboard equipment, including polyurethane cushion, board surface, sole, and insole (Determan et al., 2009; Liu et al., 2018). These structures are important in the process of cushioning and absorbing energy. Future research could therefore optimize these parts and improve the technology of the equipment to reduce the risk of injury to the skateboarder.

There are some limitations to this study. First, the bone and cartilage of the foot were fused in this study to simplify the model upon considering the complex computational environment of multibody coupling. At present, several studies have shown that it is feasible to use fused bone for research (Ishii et al., 2014; Nouman et al., 2021), but there are also studies showing that the use of fused bone will increase the contact pressure. Therefore, the multibody coupling model used here must be refined (Wang et al., 2014). It is necessary to utilize sports biomechanics experimental equipment to thoroughly analyze the dynamic and kinematic data of skateboards. Further refinement of the multibody coupling model is necessary, including refinements to the bones, ligaments, and fascia. This study solely simulated the characteristic moments of the landing buffer stage, so subsequent studies are needed to restore the ollie action moments of the entire maneuvre.

## 5 Conclusion

This study presents effective simulations and analyses of the characteristic moments of static standing and landing in skateboarding. The multibody coupling model of a skateboard based on FE analysis provides a tool to optimize the skateboard shoes and skateboard. During landing, the metatarsal descent increases gradually from the outside to the inside, and there is a tendency for the transverse arch to disappear. When maximum cushioning is achieved, a greater force is applied to the soft tissues of the medial forefoot as well as the second, third, and fourth metatarsals. Skateboard equipment must therefore provide increased cushioning of the forefoot to reduce the risk of injury to the forefoot during landing.

## Data Availability

The original contributions presented in the study are included in the article/Supplementary Material; further inquiries may be directed to the corresponding author.
