# Supplementary material for: Cushioning mechanism of the metatarsals during landing for the skateboarding ollie maneuver
Source: Front Bioeng Biotechnol. 2024 Apr 22;12:1382161. doi: 10.3389/fbioe.2024.1382161 (PMC11070832; doi:10.3389/fbioe.2024.1382161)
Supplement: Supplementary file 1 [file Table1.docx]

Supplementary Material

**Table 1 - Finite element material properties of each part.**

| **Name** | **Density（*kg/m^3^*）** | **Young's modulus (*MPa*)** | **Poisson's ratio** | **Cite** | **Nodes** | **Elements** | **Mesh Quality** |
| --- | --- | --- | --- | --- | --- | --- | --- |
| Bone | 1500 | 7300 | 0.3 | (Ishii et al., 2014) | 56105 | 36092 | 0.83 |
| Soft tissue | 937 | 1.15 | 0.49 | (Song et al., 2022) | 139371 | 92902 | 0.82 |
| Skateboard | 2000 | 11000 | 0.48 | (Li et al., 2022) | 73950 | 41802 | 0.74 |
| Wheel | 1250 | 12.516 | 0.49 | (Huo et al., 2022; Wang et al., 2022) | 20832 | 18080 | 0.69 |
| Shoe sole | 2300 | 8 | 0.47 | (Qiu et al., 2011) | 26856 | 17145 | 0.79 |
| Upper | 998 | 10 | 0.42 | (Ishii et al., 2014) | 30717 | 19503 | 0.73 |
| Insole | -- | 5 | 0.4 | (Lewis, 2003) | 36764 | 22446 | 0.77 |
| Ground | 5000 | 17000 | 0.1 | (Zhu et al., 2023) | 23448 | 12430 | 1 |

Huo, J., Li, H., He, R., Xuan, J., Zi, T., and Chake, L. (2022). Finite element analysis of mechanical properties and impact resistance of polyurethane coatings on steel structures. *Corrosion & Protection* 43(08)**,** 80-85+188.

Ishii, H., Sakurai, Y., and Maruyama, T. (2014). Effect of soccer shoe upper on ball behaviour in curve kicks. *Scientific reports* 4**,** 6067. doi: 10.1038/srep06067.

Lewis, G. (2003). Finite element analysis of a model of a therapeutic shoe: Effect of material selection for the outsole. *Bio-medical materials and engineering* 13**,** 75-81.

Li, R., Ling, Z., Mu, Q., and Zhang, H. (2022). Finite element analysis of flexural properties of composite beams connected with steel and orthogonal glulam with pulp anchor and bolt. *Chinese Journal of Wood Science and Technology* 36(03)**,** 80-89.

Qiu, T.-X., Teo, E.-C., Yan, Y., and Lei, W. (2011). Finite element modeling of a 3D coupled foot-boot model. *Medical engineering & physics* 33**,** 1228-1233. doi: 10.1016/j.medengphy.2011.05.012.

Song, Y., Sun, D., Cen, X., István, B., and Gu, Y. (2022). Individualized foot-shoe coupling finite element modeling and its application in the study of metatarsal stress. *Chinese Journal of Applied Mechanics***,** 1-10.

Wang, X., Sun, F., Dai, Z., Xia, L., and Zhang, J. (2022). Finite element analysis of polyurethane gate valve seal ring based on ANSYS. *Famen* (06)**,** 455-459. doi: 10.16630/j.cnki.1002-5855.2022.06.008.

Zhu, X., Liu, J., Liu, H., Liu, J., Yang, Y., and Wang, H. (2023). Effects of Midsole Hardness on the Mechanical Response Characteristics of the Plantar Fascia during Running. *Bioengineering* 10**,** 533. doi: 10.3390/bioengineering10050533.

**Table 2 - Load Parameter Table.**

| **Characteristic moment** | **Plantar pressure (kPa)** | **Flexion force (N)** | **Flexion force arm (mm)** | **Achilles tendon force arm (mm)** | **Achilles tendon tension (N)** |
| --- | --- | --- | --- | --- | --- |
| T1 | 42.5 | -92.06 | 166.57 | 62.1 | 246.93 |
| T2 | 160 | -830.26 | 90.97 | 62.1 | 1216.24 |
| T3 | 190 | -270 | 164.17 | 62.1 | 716.68 |

**Table 3 – Stress and strain at the metatarsals during landing.**

| **Part** | **Stress（*MPa*）** | | | **Strain（*%*）** | | |
| --- | --- | --- | --- | --- | --- | --- |
|  | **T1** | **T2** | **T3** | **T1** | **T2** | **T3** |
| MT1 | 0.286 | 13.928 | 17.549 | 0.392 | 19.085 | 24.048 |
| MT2 | 0.885 | 18.215 | 22.994 | 1.212 | 24.962 | 31.511 |
| MT3 | 1.126 | 21.032 | 26.356 | 1.548 | 28.836 | 36.136 |
| MT4 | 0.607 | 23.211 | 29.161 | 0.832 | 31.803 | 39.956 |
| MT5 | 0.127 | 15.317 | 20.409 | 0.175 | 20.984 | 27.961 |

**Table 4 - The torsion angle and the displacement in the sagittal plane of the metatarsals during landing (- for toe flexion, + for dorsiflexion).**

| **Part** | **Torsion angles (°)** | | | **Displacement (mm)** | | |
| --- | --- | --- | --- | --- | --- | --- |
|  | **T1** | **T2** | **T3** | **T1** | **T2** | **T3** |
| MT1 | -0.160 | 19.639 | 26.181 | -0.045 | 0.563 | 0.997 |
| MT2 | -0.079 | 18.309 | 23.878 | -0.054 | 0.743 | 1.215 |
| MT3 | 0.054 | 14.082 | 18.451 | -0.056 | 0.777 | 1.272 |
| MT4 | 0.054 | 12.756 | 16.594 | -0.0545 | 0.769 | 1.260 |
| MT5 | 0.101 | 6.860 | 8.8643 | -0.053 | 0.358 | 0.721 |
